# Gentamicin-loaded chitosan/folic acid-based carbon quantum dots nanocomposite hydrogel films as potential antimicrobial wound dressing

**DOI:** 10.1186/s13036-022-00318-4

**Published:** 2022-12-21

**Authors:** Fahimeh Kazeminava, Siamak Javanbakht, Mohammad Nouri, Pourya Gholizadeh, Parinaz Nezhad-Mokhtari, Khudaverdi Ganbarov, Asghar Tanomand, Hossein Samadi Kafil

**Affiliations:** 1 Drug Applied Research Center, Tabriz University of Medical Sciences, Tabriz, Iran; 2 Research Center for Pharmaceutical Nanotechnology, Biomedicine Institute, Tabriz University of Medical Sciences, Tabriz, Iran; 3 Department of Reproductive Biology, Faculty of Advanced Medical Sciences, Tabriz University of Medical Sciences, Tabriz, Iran; 4 Research Laboratory of Microbiology and Virology, Baku State University, Baku, Azerbaijan; 5 Department of Microbiology, Maragheh University of Medical Sciences, Maragheh, Iran

**Keywords:** Chitosan, Wound dressing, Hydrogel film

## Abstract

**Background:**

To provide effective healing in the wound, various carbohydrate polymers are commonly utilized that are highly potent platforms as wound dressing films. In this work, novel antibacterial flexible polymeric hydrogel films were designed *via* crosslinking polymeric chitosan (CS) with folic acid-based carbon quantum dots (CQDs). To end this, folic acid as a bio-precursor is used to synthesize CQDs through the hydrothermal technique. The synthesized CQDs as a crosslinking agent was performed at different concentrations to construct nanocomposite hydrogel films *via* the casting technique. Also, gentamicin (GM), *L*-Arginine and glycerol were supplemented in the formulation of nanocomposite since their antibiotic, bioactivity and plasticizing ability, respectively.

**Results:**

The successful construction of films were verified with different methods (FT-IR, UV-Vis, PL, SEM, and AFM analyses). The GM release profile displayed a controlled release manner over 48 h with a low initial burst release in the simulated wound media (PBS, pH 7.4). Antibacterial and in vitro cytotoxicity results showed a significant activity toward different gram-positive and negative bacterial strains (about 2.5 ± 0.1 cm inhibition zones) and a desired cytocompatibility against Human skin fibroblast (HFF-1) cells (over 80% cell viability), respectively.

**Conclusion:**

The obtained results recommend CQDs-crosslinked CS (CS/CQD) nanocomposite as a potent antimicrobial wound dressing.

**Graphical Abstract:**

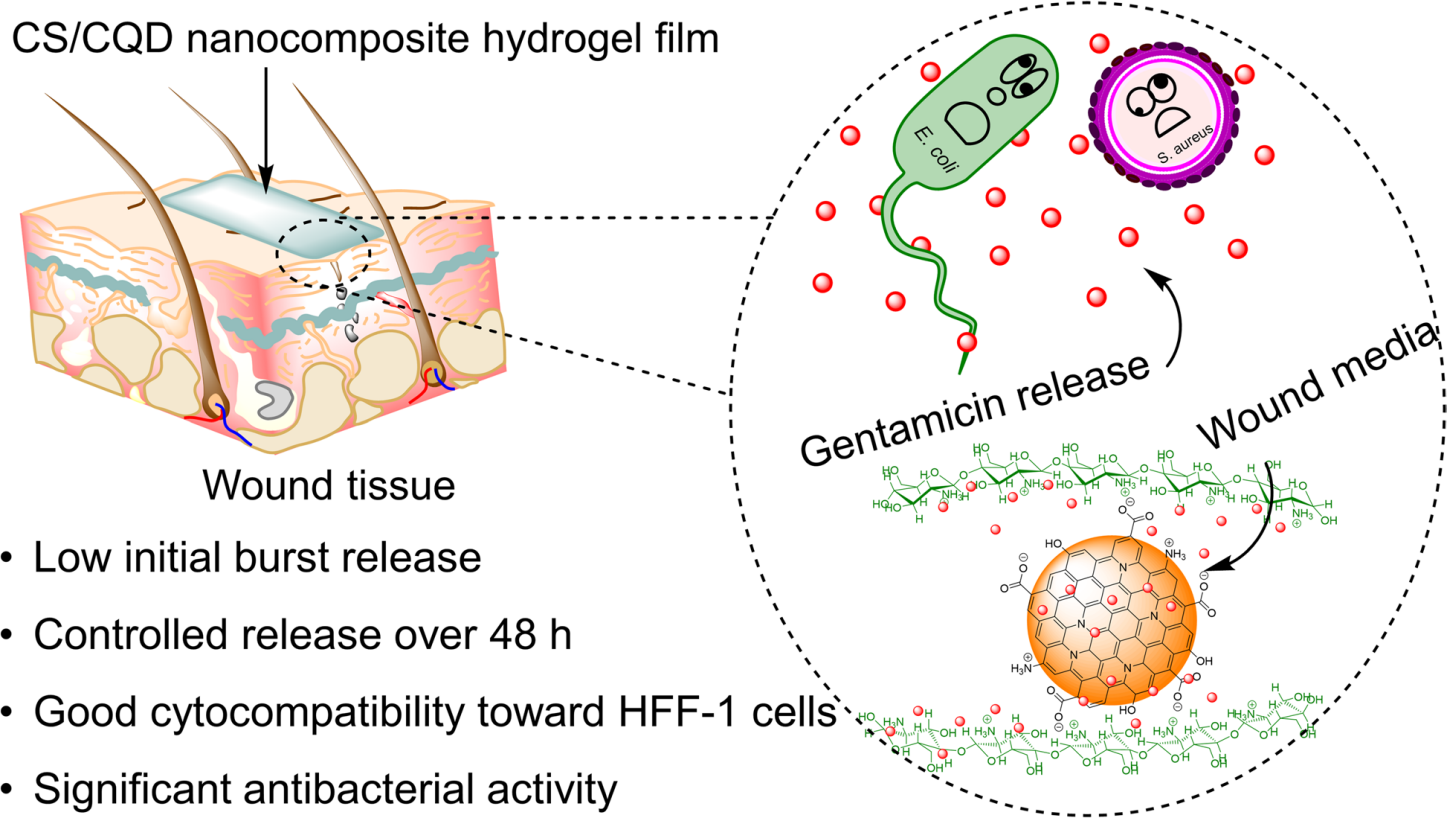

## Introduction

Skin, the widest organ of the body, does multifunctional tasks. Its injuries including all kinds of pressure sores, ulcer and burn wounds, and also other abrasive and traumatic wounds are highly costly to be cured every year. Hence, to make a good method in wound healing, lots of research on wound dressing are done [1, 2]. The infection created as a result of microorganisms such as bacteria in the injured section of the skin is one of the important challenges to wounds healing [3]. Further problems such as delay in skin recovery may occur as a result of this infection. Recently widespread range of science in different areas such as MRI contrast agents, injectable hydrogel, drug delivery, and another type of biomedical applications have concentrated on biodegradable polymers [4, 5]. For example, to provide effective healing in the wound, various biomaterials are commonly utilized [6]. Among biomaterials, carbohydrate polymers and relative hydrophilic ones are highly potent platforms as wound dressing films [7].

Chitosan, a carbohydrate polymer, has been extensively researched in different fields since its exclusive features such as biodegradability, biocompatibility, availability, etc. [8, 9]. Moreover, it can be generally employed in bio-based and biomedical applications owing to its biodegradability, biocompatibility, anti-infection, antimicrobial, and hemostatic ability properties [10–12]. On the other hand, most of these applications of chitosan are related to its hydrogel forms. The main required method for the preparation of hydrogels is the crosslinking types including chemical and physical [13–15]. While in the latter type networks are generated through intermolecular interactions, in the former, the chemical type, they are constructed *via* covalent bonding. CS hydrogel as a talented matrix platform is respected from a material point of view for its renewability, non-toxicity, biocompatibility, biodegradability, and supramolecular structure [16]. Particularly, the incorporation of different nanoparticles makes CS-based nanocomposites good candidate in biomedical fields that attracted a considerable attention [17]. Recently, the cytotoxicity of nanoparticles and crosslinking is of great challenge, particularly in biomedical applications. Therefore, it is essential to find convenient and secure crosslinkers with proper cytocompatibility and biodegradability.

In recent decades, there has been significant attention in using biocompatible nanomaterials as crosslinking agents for the construction of polymeric hydrogel [18–21]. Remarkably, fluorescent hydrogels received great attention in biomedical applications, which has been offered by the integration of individual chemical and physical characteristics of photoluminescence (PL) nanomaterials, for instance, semiconductor quantum dots, up-conversion nanoparticles, carbon quantum dots (CQDs), and luminescent metal complexes [22–25]. Because of great photo-physical and chemical features, CQDs have been regarded as a class of PL nanomaterial, absorbing remarkable and attracting interest in both technology and scientific fields [26, 27]. Likewise, they have been broadly employed in white light-emitting diodes, optoelectronic devices, catalysis, drug delivery, chemo/biosensors, bioimaging, etc. [28, 29]. Recently, a significant amount of research has been implemented on the synthesis of CQDs from bio-based materials, i.e., folic acid [30–32]. As a vital dietary component and one of the B vitamins, folic acid can well reduce the risk of some diseases [33]. It has a good potential to be adequately polymerized, condensed, and carbonized to produce N-doped CQDs without using any passivation or additive agents because of the containing rich nitrogen and functional groups of –COOH, –NH_2_, and –OH, in its structure [30].

As the above mentioned stated of art, we opted to construct innovative fluorescent hydrogel films as a potential wound dressing application based on chitosan as an easily obtainable carbohydrate. For this purpose, folic acid-based CQDs were selected as a green agent for the crosslinking chitosan because of its great biocompatibility, high surface functionality, and also controlling drug release ability. Hydrogel films of CQDs-crosslinked chitosan (CS/CQD) nanocomposite were prepared *via* a straightforward casting technique. Gentamicin (GM) is a powerful commercially available antibiotic drug prescribed commonly due to its efficacy in killing a variety of Gram-positive and negative bacteria. Also, the effect of CQDs content on the structural features and release manner of the hydrogel films was deliberate. Moreover, their antibacterial activity against different types of bacterial, in-vitro GM release, blood clotting, hemolysis, and cytotoxicity toward Human skin fibroblast HFF-1 cell lines were studied, enabling us to distinguish the ability of CS/CQD as an antibacterial film. This procedure may open a new vision in constructing a simple platform as antimicrobial wound dressing.

## Results and discussion

### CS/CQD preparation

**Fig. 1:**
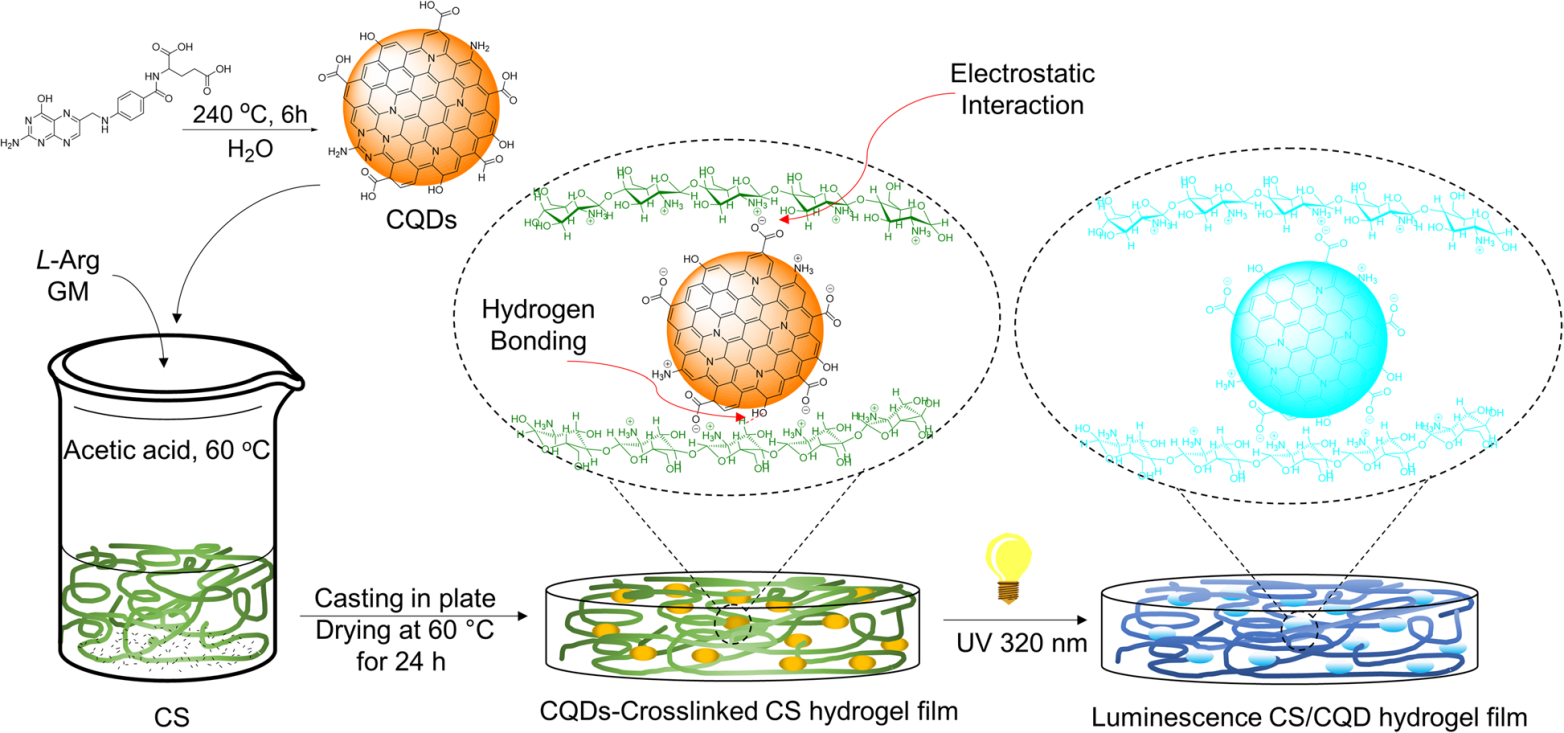
Schematic representation of the crosslinking CS with CQDs toward the preparation of CS/CQD nanocomposite hydrogel film

In this study, hydrogel films with the nanocomposite of CQDs and chitosan biopolymer were designed (Fig. 1). First, folic acid as a bio-precursor is used to synthesize CQDs through the hydrothermal technique. Next, CS/CQD nanocomposites were prepared by incorporating GM as the antibiotic agent and different concentrations of CQDs as a crosslinking agent within the CS matrix *via* the straightforward casting method. *L*-Arg and Glycerol were also added in the nanocomposite formulation since their bioactivity and plasticizing capability, respectively. The carboxylate functional groups in the CQDs structure make them potential to interact with CS by generating electrostatic interaction and hydrogen bonding with its amine and hydroxyl functional groups, respectively [20]. The prepared CS/CQD nanocomposite hydrogel films with controlling the release profile of GM could be considered as an environmentally friendly, cytocompatible and low-cost anti-infection platform.

### Characterization of the materials

**Fig. 2:**
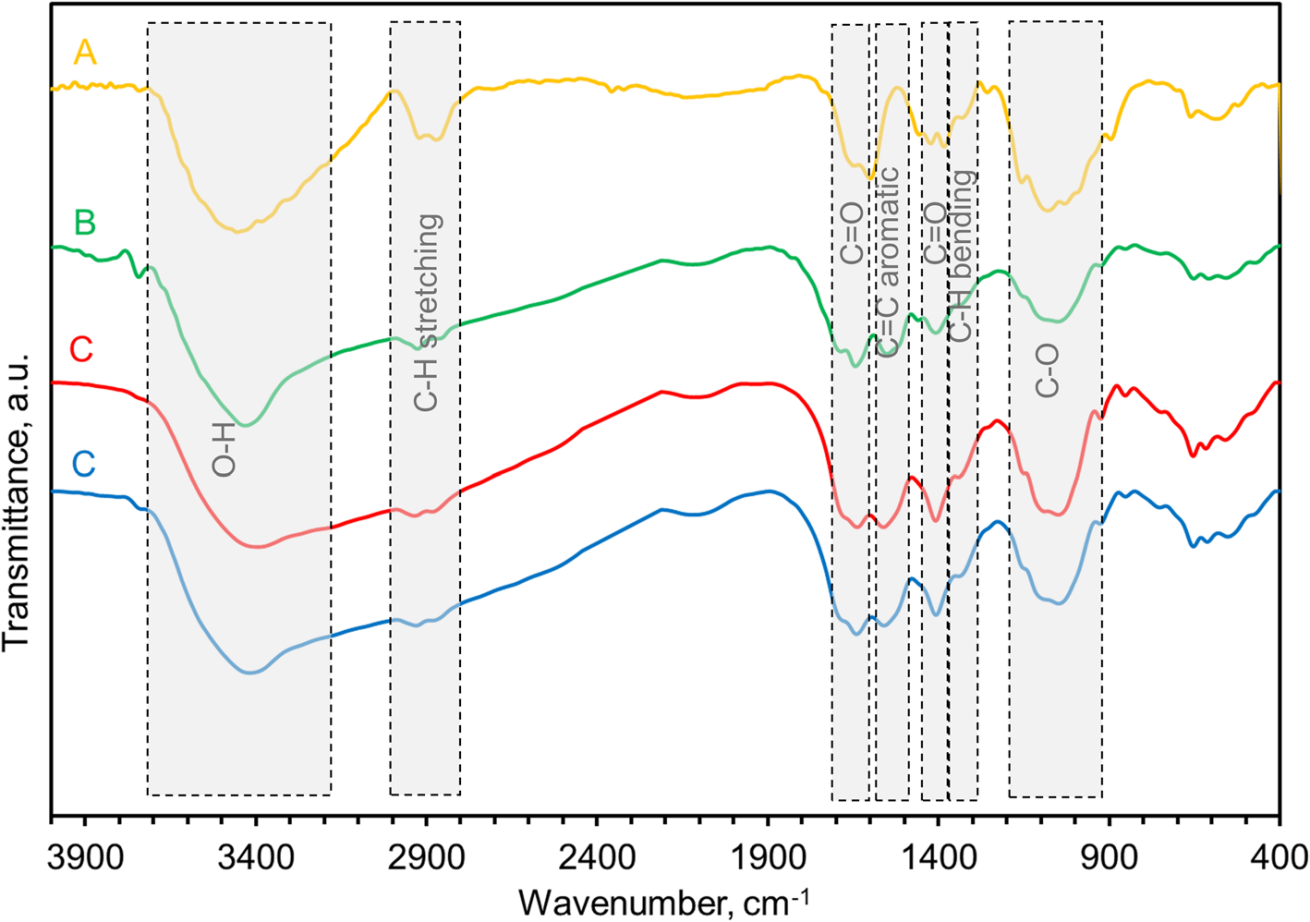
FT-IR spectra for the CS (**A**), CS/CQD 5% (**B**), CS/CQD 10% (**C**), and CS/CQD 15% (**D**)

The FT-IR spectra of CS, CS/CQD 5%, CS/CQD 10%, and CS/CQD 15% are considered to realize the successful construction of nanocomposites and also their possible interactions (Fig. 2). In the CS FT-IR spectrum, the peaks appeared at 1604 and 1590 cm^− 1^ are related to their C = O stretching vibrations of amide moieties and the N–H bending vibrations of amines, respectively. Comparison of the spectrum of CS and CS/CQD nanocomposite hydrogel films shows an appearance of a new peak at 1520 cm^− 1^ ascribed to the skeletal vibration of aromatic rings. Also, the peaks at the range of 3200–3600 cm^− 1^ related to the stretching vibrations of O-H become wider in the spectrum of CS/CQD that can be due to the H-bonding correlations between CS and CQDs. The appearance of these alterations can confirm the presence of CQDs in the CS polymeric network [20].

**Fig. 3:**
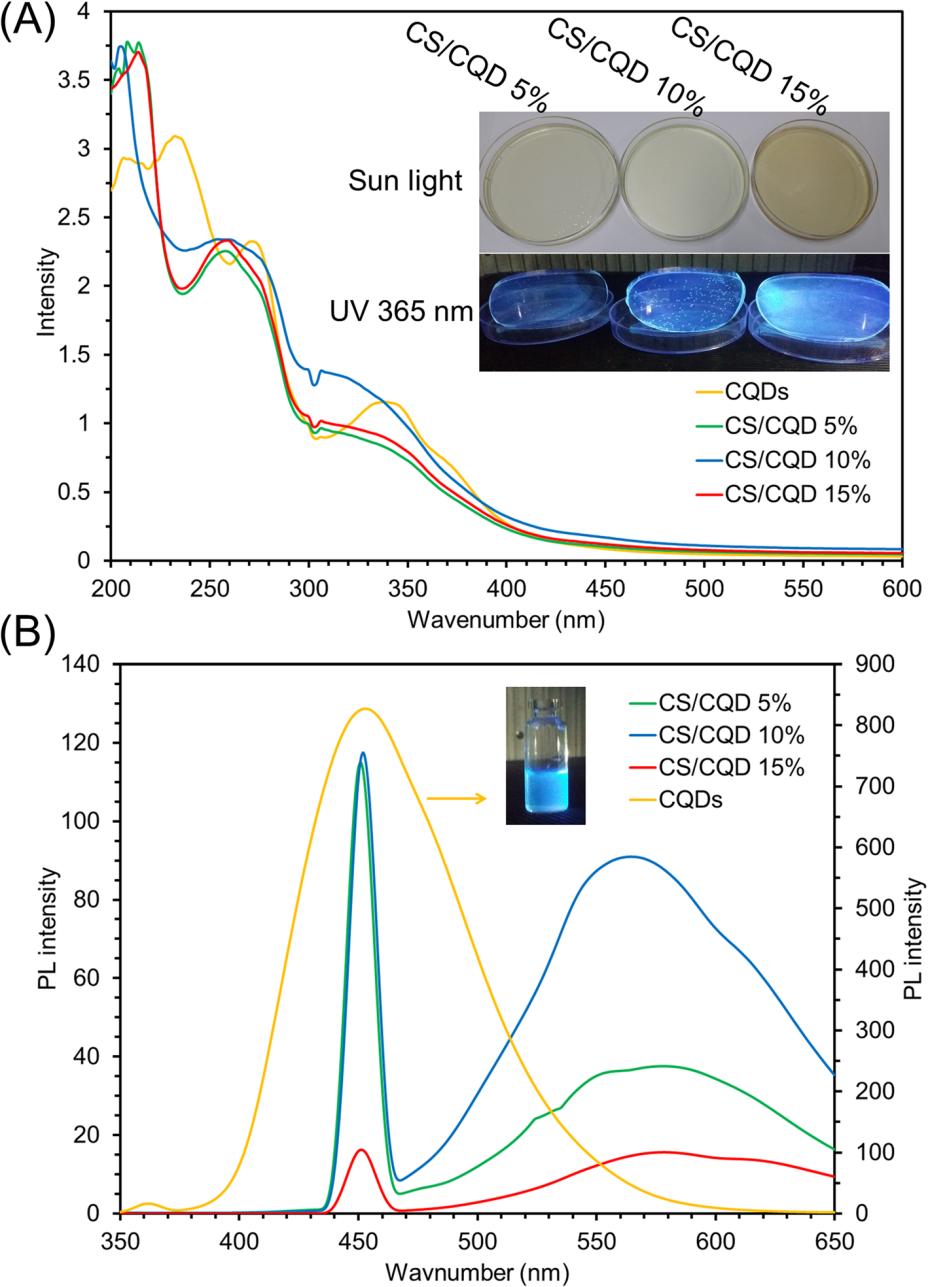
UV (**A**) and PL (**B**) spectra for the CQDs (**A**), CS/CQD 5% (**B**), CS/CQD 10% (**C**), and CS/CQD 15% (**D**) with the photographic digital image under sun and UV lamp (365 nm)

The optical and luminescent properties of the prepared films were evaluated using UV–vis and PL spectroscopy techniques, respectively. Figure 3A displays the desperation UV–vis spectra of CQDs, CS/CQD 5%, CS/CQD 10%, and CS/CQD 15%. A characteristic π-π* transition absorption peak related to the aromatic sp^2^ domains, a typical n-π* transition absorption, and a long tail widening into the visible range are observed for CQDs [34]. After the incorporation of CQDs within the CS polymeric matrix, a steady blueshift was observed for the n-π* transition absorption peak in UV–vis spectra of CS/CQD nanocomposite. This is probably due to electrostatic interaction between CQDs and CS and consequently variation in the electronic transitions of π-π* and n-π* by refilling or depleting the CQDs valance bands. The maximum emission wavelength for CQDs was observed at 450 nm (Fig. 3B). Due to the high ultimate fluorescence feature of folic acid-based CQDs, it is triggered in composition with CS, which can visibly induce in the CS/CQD nanocomposites (photographic images under UV 365 nm lamp in Fig. 3B). Also, another emission wavelength can be seen in the PL spectra of CS/CQD nanocomposites at about 570 nm. For these observations, some reasons have been reported such as the self-absorbing, filter effect, inner photoluminescence reabsorption, internal or external transformations phenomena, partial quenching, extinction effect, and intersystem transitions [35]. As the exact mechanism is not completely known, we recommended further investigations to fully address this subject.

**Fig. 4:**
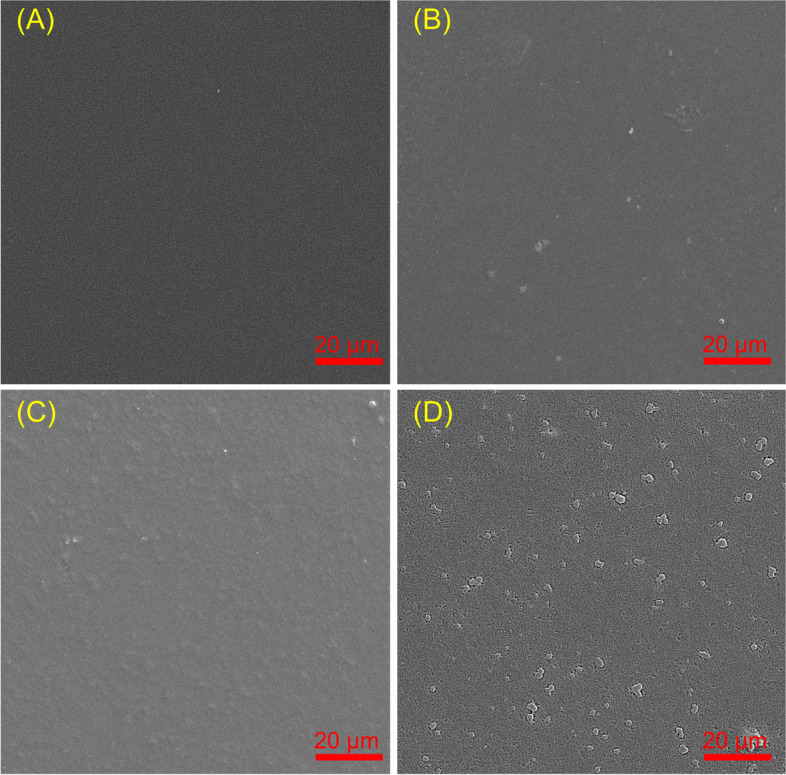
SEM images of CS film (**A**), CS/CQD 5% (**B**), CS/CQD 10% (**C**), and CS/CQD 15% (**D**) hydrogel films

**Fig. 5:**
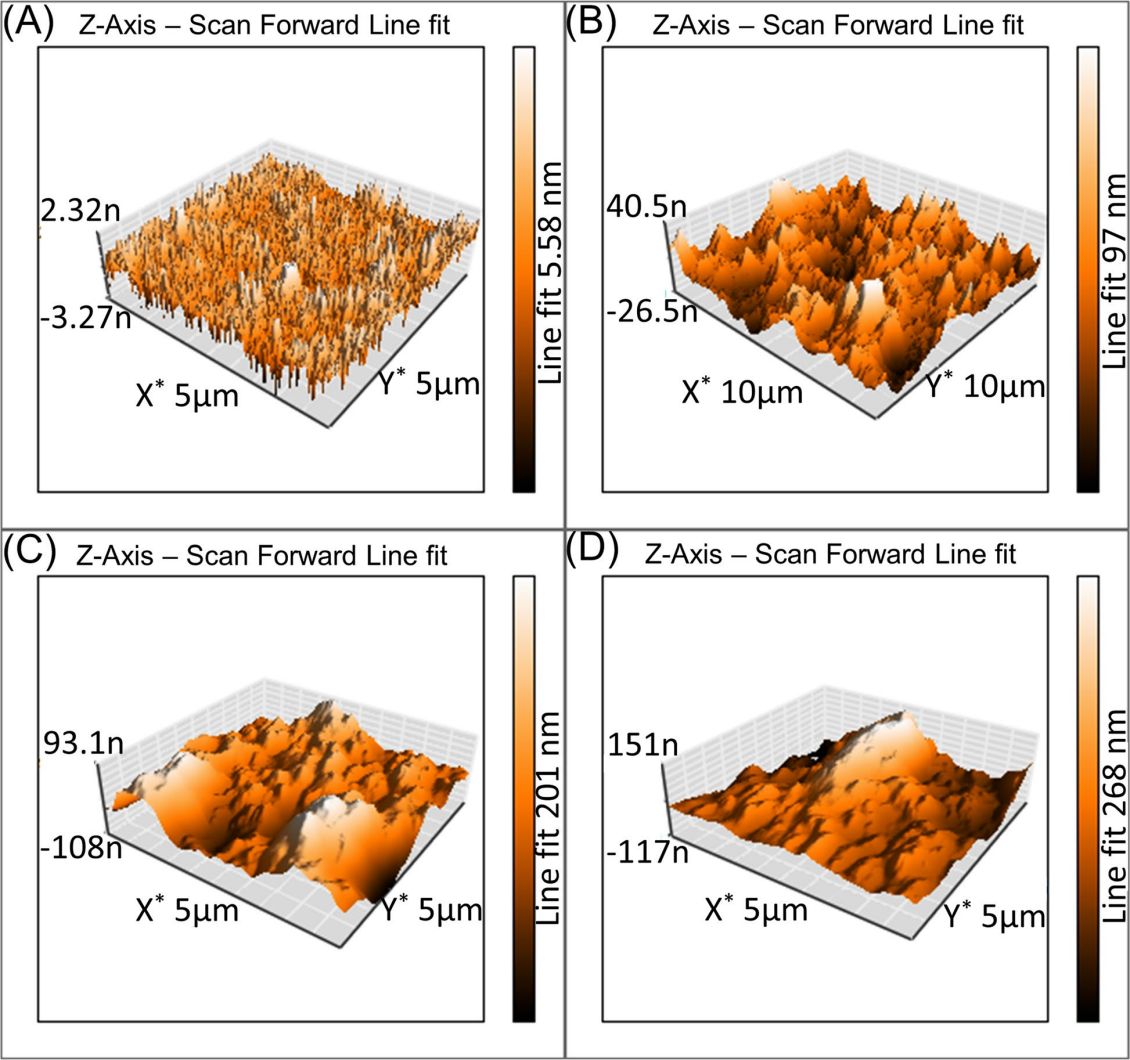
AFM images of CS (**A**), CS/CQD 5% (**B**), CS/CQD 10% (**C**), and CS/CQD 15% (**D**)

Figure 4 displays the SEM images of the CS film and CS/CQD with different concentrations of CQDs from 5 to 15%. From the SEM images, all films have a uniform surface morphology; however, by increasing the amount of CQDs in the nanocomposite, the surface morphology of the nanocomposite was slightly changed to be rough. On the other hand, there are visible the morphology of hydrogel films be a little altered by increasing CQDs (Fig. 5, AFM images). This contribution may be attributed to the nanoparticles aggregation by rising their concentration in the nanocomposite hydrogel films.

**Fig. 6:**
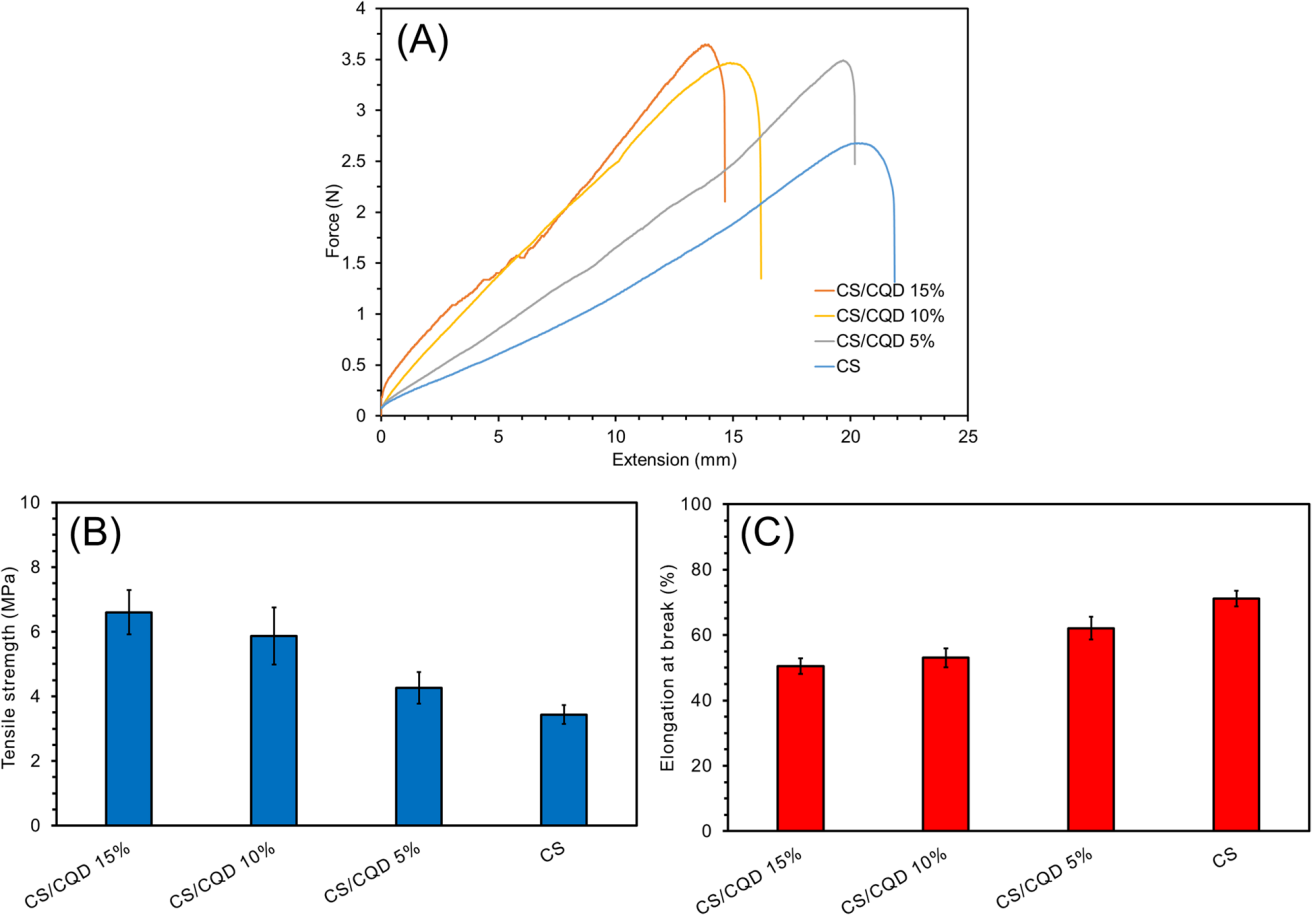
Stress (**A**) and elongation at break (**B**) of the CS and CS/CQD with different CQDs content

The CS film and CS/CQD 5%, CS/CQD 10%, and CS/CQD 15% showed a tensile strength of 3.43, 4.26, 5.86, and 6.60 MPa, respectively (Fig. 6a). The CQDs incorporation in the CS network increased the tensile strength of films due to its crosslinking capability. Comparing the tensile strength value of CS/CQD with different CQDs concentrations showed an enhancement with rising the CQDs content. To consider the flexibility of the films, their elongation at break values was also considered. This value for CS film without CQDs is higher than that of CS/CQD nanocomposites (Fig. 6b). Whereas the integration of CQDs reduced the flexibility of the samples, they were suitable to allocate the operational force for standing over a variety of bodies or wound surfaces [36, 37].

**Fig. 7:**
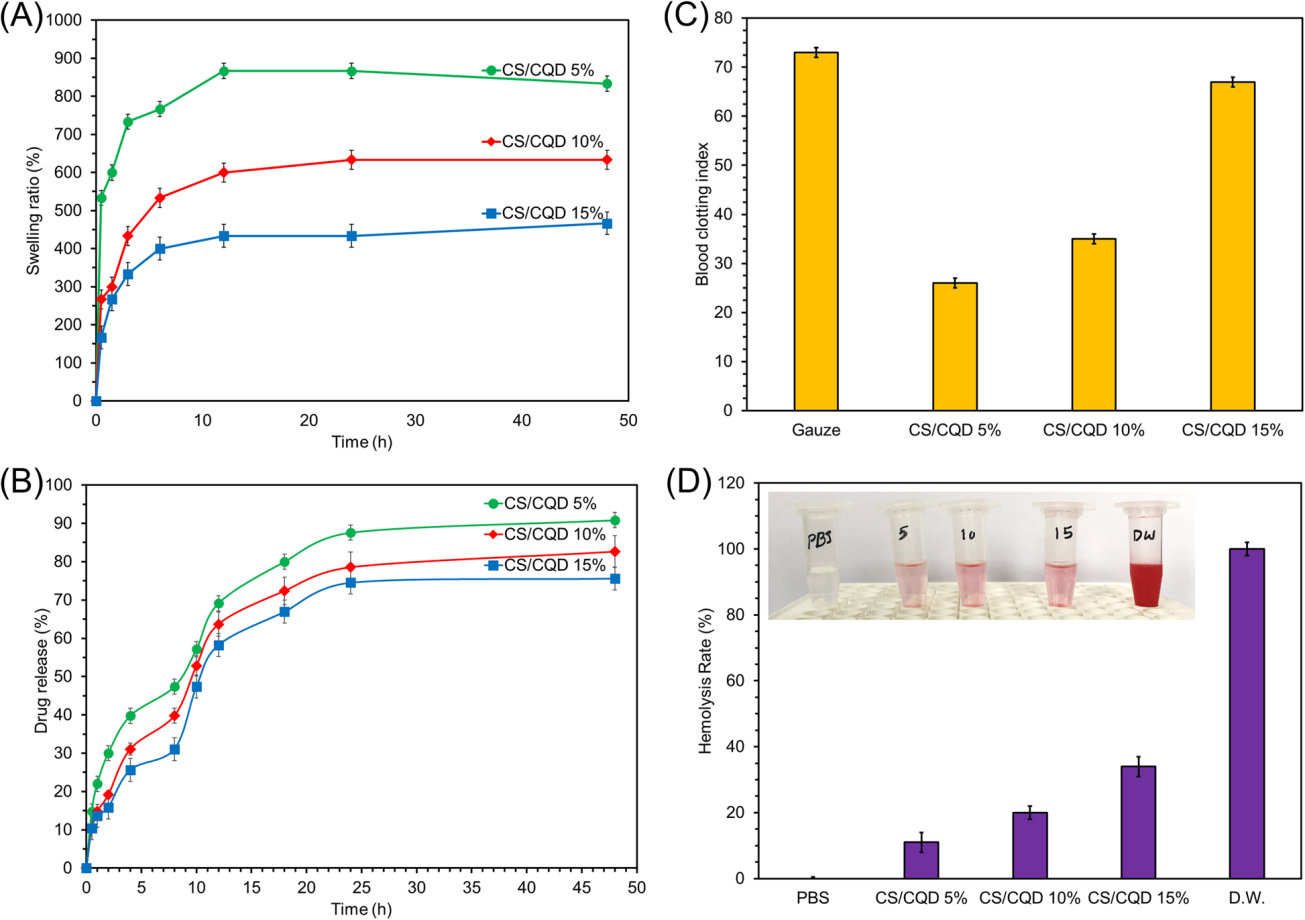
The swelling ratio in PBS (pH 7.4) at 37 °C for the CS and CS/CQD nanocomposite hydrogel films (the average of three measurements was reported) (**A**). GM release from the prepared CS and CS/CQD nanocomposite hydrogel films in PBS (pH 7.4) at 37 °C (**B**). The results of the whole blood clotting index (**C**) and hemolysis rate (**D**) of CS/CQD nanocomposite hydrogel films after incubation with human blood and human red blood cells (erythrocytes), respectively

Figure 7A demonstrates the swelling performance of the films in PBS (pH 7.4) at 37 °C. It was shown that the swelling ratio was noticeably raised with immersing time. The incorporating CQDs in the polymeric matrix of CS diminishes the swelling ratio. This can be due to the more crosslinking of the CS biopolymer and rigidity of the hydrogel network [21]. From the swelling results, the prepared nanocomposite hydrogel films can absorb wound moisture and release the loaded drug.

### In-vitro drug release study

Figure 7B shows the GM release chart of the CS and CS/CQD nanocomposite hydrogel films achieved in PBS (pH 7.4) at 37 °C. GM release chart exhibited a controlled and continued release over 48 h with a lower initial burst release. According to the literature [38], this can depend on the diverse interaction between CQDs and GM molecules, for example, electrostatic interaction, π-π stacking, and hydrogen bonding, which is in agreement. Therefore, the content of CQDs in the polymeric CS matrix impacts the release manner of GM. It was shown a diminish in GM release with increasing the CQDs concentration in the nanocomposites. Not only this is related to the increasing the possible interaction between CQDs and GM drug, but also the network rigidity of hydrogel affects this contribution, which is confirmed in the section of materials characterization. The results of release study are comparable with the similar reports on the GM delivery with respect to the initial burst release [39, 40]. Therefore, the prepared nanocomposite films are suitable platform to release a certain dosage of GM to the wound tissue and prevent any possible side effects of its exploded initial release.

### Blood clotting and hemolysis studies

A key factor related to the wound dressing based on biomaterials is the blood clotting ability. The results of the blood clotting study are represented in Fig. 7C. According to the results, the blood capability of nanocomposite films with raising the concentration of CQDs has enhanced. The blood clotting ability of CS/CQD 15% showed a higher blood clotting ability than that of other films, as well as it is comparable with commercial Gauze. Probably, this is can be related to the bioactivity of folic acid-based CQGs. Obtained results reveal the haemostatic potential of the prepared CS/CQD nanocomposite as the wound dressing applications [41].

In the normal human blood, the erythrocyte rate is ~ 50% and its interaction with blood-contacting platforms is critical to consider the hemoglobin release. The hemolytic effects were respectively increased with hemolysis rate of 11%, 20%, and 34% with raising the concentration of CQDs in the formulation of CS/CQD nanocomposites (CS/CQD 5%, CS/CQD 10%, and CS/CQD 15%), Fig. 7D. Also, the optical image inside Fig. 7D displayed the supernatant of treated films with red blood cells (hemoglobin), which is meaningful in comparison with negative control (PBS). Therefore, the film with low CQDs content (CS/CQD 15%) is the desired candidate for employment as a wound application [42].

### Cytotoxicity study

**Fig. 8:**
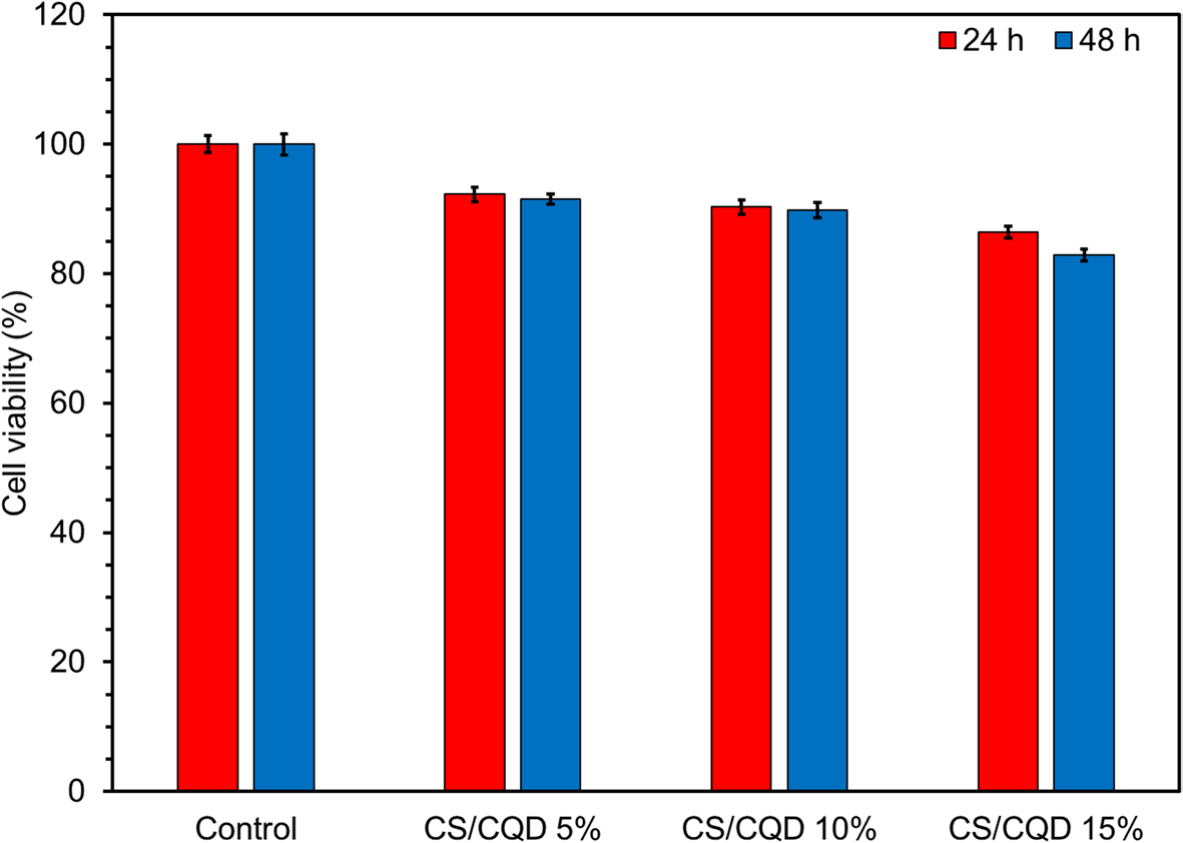
Cell viability of HFF-1 cell lines on treating with CS/CQD nanocomposite hydrogel films after different incubation time

MTT test was carried out to investigate the cytotoxicity of the CS and CS/CQD hydrogel films against HFF-1 cell lines (Fig. 8). The cell viability of samples has remained over 80% even after 48 h. On the other hand, the result shows the cytotoxicity of hydrogels was modestly increased with treated time and the content of CQDs in the nanocomposite. This desired cytocompatibility of the CS/CQD may be related to the good cytotoxicity nature of CS and folic acid-based CQDs reported in the literature [30, 43, 44]. From the results, the constructed CS/CQD films could potentially to suggest a safe wound dressing platform.

### Antimicrobial activity

**Fig. 9:**
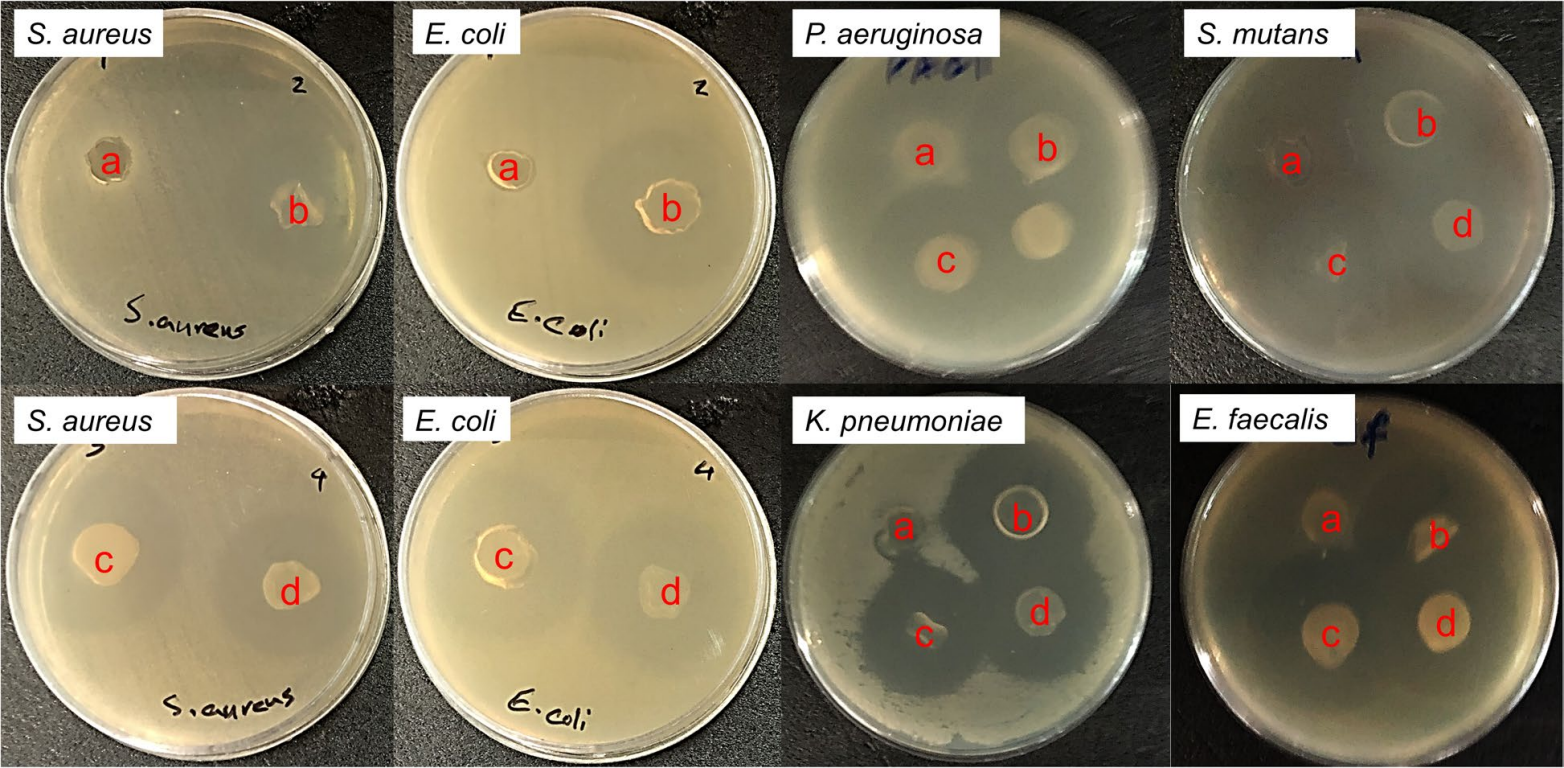
Images of the antibacterial inhibition zones for CS (a), CS/CQD 5% (b), CS/CQD 10% (c), and CS/CQD 15% (d)

To explain the antimicrobial performance of the CS/CQD nanocomposites, their activity was studied toward *E. faecalis*, *P. aeruginosa*, *S. mutans*, *S. aureus, K. pneumoniae*, and *E. coli* bacteria *via* the disc diffusion procedures. Figure 9 displays that all nanocomposites show significant antibacterial activity compared to CS film as a control sample. The antibacterial performance of constructed CS/CQD nanocomposite hydrogel films is similar due to a low difference between the GM release of samples over 24 h (Table 1). Among different bacterial strains, *E. coli* shows a lower resistance against nanocomposite. The results of our present work are comparable with the similar hydrogel films in the literatures [39, 45–47].

**Table 1:** Inhibition zones (cm) of the tested films toward different bacteria

Bacteria		*E. coli*	*S. aureus*	*E. faecalis*	*P. aeruginosa*	*S. mutans*	*K. pneumoniae*
Entry	Sample						
a	CS	0.8 ± 0.1	0	0	0	0	0
b	CS/CQD 5%	2.7 ± 0.1	2.4 ± 0.1	2.4 ± 0.1	2.1 ± 0.1	2.5 ± 0.1	2.5 ± 0.1
c	CS/CQD 10%	3.0 ± 0.1	2.5 ± 0.1	2.4 ± 0.1	2.6 ± 0.1	2.3 ± 0.1	2.3 ± 0.1
d	CS/CQD 15%	2.7 ± 0.1	2.4 ± 0.1	2.3 ± 0.1	2.6 ± 0.1	2.3 ± 0.1	2.2 ± 0.1

## Conclusion

This study deals a green, simple and economical protocol to prepare an antimicrobial film with great efficiency based on chitosan (CS) hydrogel crosslinked with folic acid-based carbon quantum dots (CQDs) and plasticized by glycerol. Gentamicin (GM) and *L*-arginine (*L*-Arg) as antibacterial and bioactive agent are also incorporated in the nanocomposite formulation. The synthesizing CQDs was successfully performed through the hydrothermal technique. The unique functional groups of folic acid-based CQDs, i.e., carboxylate moieties, make it suitable for crosslinking CS *via* electrostatic interaction. For the construction of hydrogel films, this type of processing offers feasible properties since the availability of starting materials and applications of green approaches, i.e., simple casting and hydrothermal methods. The incorporation of CQDs impacts the polymeric network rigidity (improved the stress and elongation at break values about 2 MPs and 20%, respectively) as it acts as a crosslinker agent. The successful construction of nanocomposites was confirmed with different techniques (FT-IR, UV-Vis, PL, SEM, and AFM analyses). Gentamicin (GM) release from the hydrogel films showed a sustained and controlled release profile over 48 h with a lower initial burst release. This is because of the synergistic effect of CS and CQDs in controlling the release manner in which they enhance the diversity of probable interactions between carrier and GM molecules. Outstandingly, among the nanocomposite, CS/CQD 15% shows the desired blood clotting (about 70%), haemostatic potential (about 34%), notable antibacterial activity toward different bacterial strains (about 2.5 ± 0.1 cm inhibition zones), and cytotoxicity against Human skin fibroblast (HFF-1) cell lines (over 80% cell viability). We believe that the present protocol may receive great interest to prepare nanocomposite hydrogel films as wound dressing bandages through the green protocol to control microbial infection in wound exudate.

## Methods

### Materials

Chitosan (99.5%, medium molecular weight), acetic acid (98%), folic acid, L-Arg, Glycerol (99.5%), and all other chemical were procured from Merck Co. All biological agents were supplied from Sigma Aldrich, USA.

### Characterization and analysis

UV–vis absorption spectra of the samples were gained with a spectrophotometer (Shimadzu, model 2450). An FTIR spectrometer (Bruker Instruments, model Aquinox 55, Germany) was used to obtained infrared spectra of the samples. Moreover, the surface morphology of the samples was studied using a scanning electron microscope (SEM; LEO, 1430VP) after coating the samples with gold films operating at 5 kV and atomic force microscopy (AFM, FLEX, Nanosurf, Swiss) were used. The fluorescent properties of the films were recorded through a spectrofluorometer (FP-6200, JASCO). Determining the elongation at break and tensile strength of the samples was possible by using a universal testing machine (Zwick/Roell, Model, Z010 Germany).

### Synthesis of N-doped CQDs

The N-doped CQDs were synthesized using FA as a bio-precursor through a hydrothermal technique [30]. A well-distributed solution of FA was prepared by dissolving 10 mg of FA in 15 mL ultrapure water and sonicated for 5 min. Subsequently, it is treated in an oven for 8 h at 200 °C for carbonization. As a result of this reaction, a clear light yellow-brown solution was attained. Finally, to remove the precipitate, the solution was centrifuged for 15 min at 11,000 rpm. The supernatant was kept at 0 °C in dark for further use.

### Preparation of CS/CQD/GM hydrogel films

1% (w/v) homogenous solution of chitosan was prepared by dissolving a certain amount of chitosan powder (1.000 g) in 0.1% (w/v) acetic acid solution and stirred for 1 h at ambient temperature. Certain amounts of CQDs (0.050, 0.100 and 0.150 g) were dissolved in 20 mL distilled water to prepare 5%, 10%, and 15% solutions (weight% of CQDs to CS content), respectively. Afterward, CQDs and GM (0.010 g) were separately added to the CS solution and stirred until dissolving, followed by the supplementation of *L*-Arg (0.010 g) to the prepared mixture. Then, as a plasticizer, glycerol (0.5% w/v) was added to the prepared solutions and stirred for 4 h at 50 °C. The formed homogeneous and transparent paste (25 mL) was cast onto a polystyrene plate with a 10 cm diameter and cured at 60 °C for 24 h.

### Swelling study

The in-vitro swelling examination was performed at physiological temperature (37 °C) using phosphate-buffered saline (PBS, pH 7.4) solution. Samples (CS/CQD/GM hydrogel films) were weighed (Wi) and socked in 20 mL of the buffered solution. To determine the swelling ratio, samples were taken out at predetermined time intervals and then weighted (Wt) after removing the surface water using a filter paper. The following equation (Eq. 1) was used to determine the swelling ratio:

1 $$Swelling\;ratio=\frac{Wt-Wi}{Wi}$$

### Drug release study

According to the reported procedure [47], to investigate the drug release profile, each hydrogel film (10 mg) was immersed in phosphate buffered media (10 mL, PBS, pH 7.4) to simulate the physiological microenvironment. Afterward, an aqueous solution (3 mL) of released media was withdrawn and replaced with fresh buffered media at a certain time break. Standard calibration curve for GM at 270 nm used to attained the concentration of drug in the released media. The release profile was considered by the Eq. 2:

2 $$Drug\;release=\frac{the\;amount\;of\;released\;drug}{the\;amount\;of\;loaded\;drug}\times100$$

### Blood clotting

Reported protocols demonstrate the blood clotting assay performance [42]. Sodium citrate as an anticoagulant stabilized the fresh human blood. Initially, all sliced films with 10 mm diameter are located into the 12 well-plates. To pre-warm the films, the plate was placed into an incubator for 15 min at 37 °C. Subsequently, stabilized blood (200 µL) was gradually dropped on the films. Here, the controls group were well with gauze and no sample. Then, to initiate the blood-clotting 40 µL of CaCl_2_ solution (0.2 M) was added into the blood samples. After incubation for 15 min at 37 °C, the red blood cells are trapped in the clotting. Subsequently, to hemolysis the blood cells, 6 mL of deionized water was added to each well. After 2 min gentle shaking, 2 mL of media was withdrawn and centrifuged for 2 min at 1500 rpm. The absorbance of the supernatant was considered at 540 nm in water using ELISA-reader (BioTech). The blood clotting index (BCI) was measured with the following Eq. 3 for various samples.

3 $$\text{B}\text{C}\text{I}= \frac{ \text{O}\text{D}\text{S}\text{a}\text{m}\text{p}\text{l}\text{e}}{\text{O}\text{D}\text{C}\text{o}\text{n}\text{t}\text{r}\text{o}\text{l}}$$

where OD_sample_ and OD_Control_ were absorbances of blood that had been in contact with samples and without the sample, respectively.

### Hemolysis rate assay

Hemolysis assay was carried out according to the reported procedure with a little modification [42]. At first, the supernatant (plasma) stabilized human fresh blood was taken out by centrifuging. To eliminate lysed hemoglobin, the pellets (human red blood cells) were rinsed 3 times with PBS (pH 7.4). The pellets were immersed in PBS (diluted ten times) and blended with 1 mL PBS having CS/CQD 5%, 10% and 15% and incubated for 60 min at 37 °C. The positive control with 100% hemolysis was obtained by double-distilled water, and the negative control with 0% hemolysis was induced with PBS solution. The incubated samples were centrifuged at 3500 rpm for 10 min. The supernatant was taken to the 96-well plates and their absorbance was measured using a spectrophotometer at 545 nm. The percent of hemolysis rate was determined using the following Eq. 4.

4 $$\text{Hemolysis Rate}\left(\%\right)=\frac{(\text{Abs sample}\;-\;\text{Abs PBS})}{(\text{Abs water}\;-\;\text{Abs PBS})}\times100$$

### Cell culture

Human skin fibroblast (HFF-1) cells were attained from Pasteur Institute of Iran (Tehran, Iran) and cultured in Dulbeccom modified Eagle medium (DMEM, high glucose formulation; Gibco BRL containing 100 mg/mL streptomycin, 100 unit/mL penicillin and 10% (v/v) fetal bovine serum. Cells were seeded in triplicate into 96-well plates at a density of 1 × 10^4^ cells/well and were incubated at 37 °C for 24–48 h (incubator containing 5% CO_2_) and used in tests when they achieved 80% confluence at logarithmic growing phase.

### Cell viability assay

The biocompatibility and the cytotoxicity tests of the nanocomposite hydrogel films were assessed by MTT assay. The films were sterilized with ethanol 70% for 5 min afterward exposure to UV irradiation for 20 min. The samples were washed 2 times with PBS (pH 7.2) and then immersed in a fresh culture medium. After 24 h, HFFF-1 cells were cultured at the density of 10^4^ cells per well into 96-well plates (including samples) in triplicate. As a control group, cells without samples were considered that only received fresh medium. To solubilize the formazan crystals, the medium was replaced with 150 µL fresh culture medium containing MTT solution (5 mg·mL^–1^) and incubated at 37 °C for 4 h. Then, the medium was taken out and DMSO (200 µL) was added to dissolve the purple-blue MTT formazan precipitate. The absorbance of solubilized formazan was recorded at 570 nm using a multi-well plate reader (Quant Bio-tek Instruments, Winooski, VT, USA) after 30 min and the cell viability was calculated with following Eq. 5.

5 $$\text{Cell viability}\left(\%\right)=\frac{\text{Mean absorbance of each group}}{\text{Mean absorbance of the control group}}\times100$$

### Antimicrobial activity

The antimicrobial performance was carried out with the agar disc diffusion method [48, 49]. To investigate antibacterial activities, the synthesized nanocomposite films were tested against six pathogens and results are presented in Fig. 9; Table 1. The bacteria strains were adjusted to 0.5 McFarland standard and followed by they were treated on the surface of agar plates. Afterward, all films were cut in a sterile condition with 8 mm diameter and were put on agar plates, and then they were incubated at 37 °C. After 18–24 h, the inhibition zones were measured.

## Data Availability

The datasets generated during and/or analyzed during the current study are available from the corresponding author on reasonable request.
